# Human Machine Interface with Wearable Electronics Using Biodegradable Triboelectric Films for Calligraphy Practice and Correction

**DOI:** 10.1007/s40820-022-00965-8

**Published:** 2022-11-15

**Authors:** Shen Shen, Jia Yi, Zhongda Sun, Zihao Guo, Tianyiyi He, Liyun Ma, Huimin Li, Jiajia Fu, Chengkuo Lee, Zhong Lin Wang

**Affiliations:** 1 Jiangsu Engineering Technology Research Center for Functional Textiles, Jiangnan University, No.1800 Lihu Avenue, Wuxi, P. R. China; 2 Department of Electrical and Computer Engineering, National University of Singapore, 4 Engineering Drive 3, Singapore, 117576 Singapore; 3 CAS Center for Excellence in Nanoscience, Beijing Key Laboratory of Micro-Nano Energy and Sensor, Beijing Institute of Nanoenergy and Nanosystems, Chinese Academy of Sciences, Beijing, 100083 P.R. China; 4 China National Textile and Apparel Council Key Laboratory of Natural Dyes, Soochow University, Suzhou, 215123 People’s Republic of China; 5 School of Material Science and Engineering, Georgia Institute of Technology, Atlanta, GA 30332-0245 USA

**Keywords:** Letter handwriting, Triboelectric nanogenerator, Biodegradable, Human–machine interface, Calligraphy practice

## Abstract

Letter handwriting, especially stroke correction, is of great importance for recording languages and expressing and exchanging ideas for individual behavior and the public. In this study, a biodegradable and conductive carboxymethyl chitosan-silk fibroin (CSF) film is prepared to design wearable triboelectric nanogenerator (denoted as CSF-TENG), which outputs of *V*_oc_ ≈ 165 V, *I*_sc_ ≈ 1.4 μA, and *Q*_sc_ ≈ 72 mW cm^−2^. Further, in vitro biodegradation of CSF film is performed through trypsin and lysozyme. The results show that trypsin and lysozyme have stable and favorable biodegradation properties, removing 63.1% of CSF film after degrading for 11 days. Further, the CSF-TENG-based human–machine interface (HMI) is designed to promptly track writing steps and access the accuracy of letters, resulting in a straightforward communication media of human and machine. The CSF-TENG-based HMI can automatically recognize and correct three representative letters (F, H, and K), which is benefited by HMI system for data processing and analysis. The CSF-TENG-based HMI can make decisions for the next stroke, highlighting the stroke in advance by replacing it with red, which can be a candidate for calligraphy practice and correction. Finally, various demonstrations are done in real-time to achieve virtual and real-world controls including writing, vehicle movements, and healthcare.

**Supplementary Information:**

The online version contains supplementary material available at 10.1007/s40820-022-00965-8.

## Highlights

- A wearable triboelectric nanogenerator (denoted as CSF-TENG) is designed using biodegradable and carboxymethyl chitosan-silk fibroin (CSF) film.
- In vitro biodegradation of CSF film is performed through trypsin and lysozyme. 63.1% of CSF film is removed by trypsin and lysozyme after degrading for 11 days.
- An intuitive writing system is designed by CSF-TENGs-based human-machine interface to promptly track writing steps, highlight the stroke in advance, and access the accuracy of letters.

**Supplementary Information:**

The online version contains supplementary material available at 10.1007/s40820-022-00965-8.

## Graphical abstract

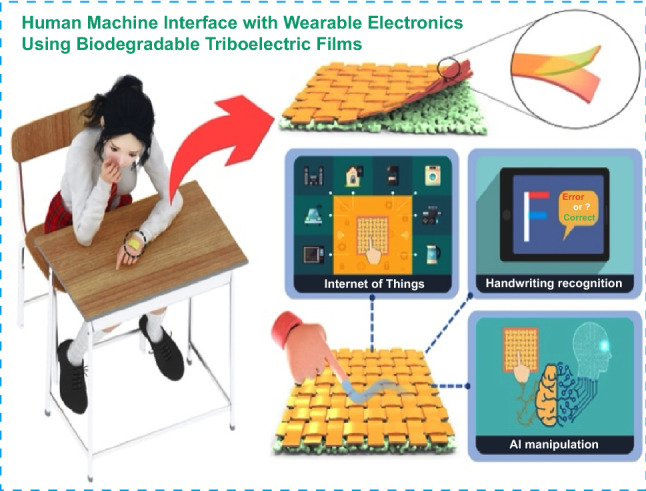

## Introduction

Wearable electronics have experienced development and progress in the past decades because of their significant contributions to various fields [1–6]. Wearable electronics components can be coated on the human body or skin to monitor human healthcare such as respiration monitor [7], body temperature [8], and safety protection [9, 10]. Furthermore, it has emerged recently toward flexible and portable devices for motion monitoring, healthcare, and human–machine interfaces (HMIs) [11–15]. Thus, the recent advancement of wearable electronics based on HMIs has sparked the digitalization of human activity through analyzing data generated by tiny movements and endowing it with wide application such as accurate recognition, gestures [16], and communication [17, 18].

Internet of things (IoT) consists of a series of devices that are interconnected over the internet or other communication networks. It has shown significant potential in activity recognition, intelligent monitoring, and real-time response systems [19–22]. Ultrafast expansion and deployment of semiconductor technology enable cost-effective means for wireless network interconnectivity between countless sensors and processors, stimulating visible development in the HMIs [23–25]. Accordingly, the burgeoning HMI fosters an imperative demand for intelligent sensors that provide a key connection between humans and machines, allowing for more effective but simpler routes to realized more required works [26–29]. Under this circumstance, huge and widely dispersed electronic devices such as wearable electronics/photonics devices are expected to be interconnected wirelessly with IoT, providing comprehensive real-time surveillance on our destinations and actions [30–33]. Hence, wearable electronics are undergoing an explosive development for human–machine interaction in IoT applications [34–36].

However, the remarkable advancement of wearable electronics necessitates the widespread use of mobile power sources, which in many situations are still traditional devices with limited lifetimes [37–40]. Thus, significant effort has been devoted to the exploration of wearable devices that can convert wasted mechanical energy to electrical signals [41–44]. Triboelectric nanogenerator (TENG) was established in response to this requirement. TENG acts as an innovation of energy collection technology that noticeably stands out from its virtues and unique traits because of enormous breakthroughs in energy-harvesting nanotechnology [45–48]. TENG-based HMIs are experiencing extensive and flourishing development, and they are used in almost all aspects of our lives for wearable sensor, medical monitoring, and energy conversion [49–52]. Thus, TENG-based HMIs exhibit many of the numerous merits (for example, low-cost, self-powered, and easy electric circuits etc.) and have continued to emerge in various fields, such as tracking identification, error information amendment, and human–computer interaction [43, 53–56]. For instance, it is reported a wearable triboelectric sensor for gait analysis and motion harvest to improve the intelligence of the robot-aided lower-limb and waist rehabilitation [57]. M. Zhu et al. intergated triboelectric sensors with an exoskeleton system to capture and project various motions of human and robotic arm [58]. C. Li et al. described a badge-reel-like stretch sensor based on TENG to monitor the change of spinal shape, illustrating application in daily spinal monitoring and physical rehabilitation training [59].

In past few years, TENG-based HMIs have been developed to track a trajectory pattern or identify letters leading to a real-time response and sustainable monitoring [60–63]. For example, W. Zhang et al. displayed a triboelectric sensor for handwriting signature identification [64]. X. Ji et al. constructed a triboelectric electronics based HMI for analyzing writing signal pattern and letters fingerprint. Further, by combining machine learning, the recognition accuracy of letters fingerprint is calculated [61]. Although the interactive communication between human and machines is achieved through the fundamental letter identification in the above-mentioned works, complex writing process hasn’t been realized by only depending on output signal or pattern identification. Particularly, calligraphy is of great importance in personal development because it is intimately associated with personal behavioral features, and is an essential part of civilian applications. Besides, traditional calligraphy boards, which are used to writing letter daily only provide writing rather than detailed monitoring, and do not meet the requirements of letters practice and correction. Thus, the real-time and sustainable monitoring of writing steps is crucial feature for practicing or correcting the aesthetic of letters and even its accuracy. Additionally, traditional wearable electronics posses low biodegradability and impermeability, even harmful materials to environment. Hence these factors restrict their applications. Notably, wearable TENG with biodegradability for letter handwriting has not yet been reported.

Here, HMI-enabled wearable electronics based on a triboelectric mechanism are constructed to achieve an intelligent, highly accurate, and real-time response writing system and carboxymethyl chitosan-silk fibroin-TENG (CSF-TENG) with a contact-separation mode fabricating the portable electronics, which uses carboxymethyl chitosan-silk fibroin (CSF) film as friction electrification material. CSF film is prepared by crosslinking carboxymethyl chitosan and silk, resulting in biodegradability, flexibility, and softness. Weight loss and UV–visible absorption change are used to investigate the biodegradation behavior of CFS film by trypsin and lysozyme. The impact of silk content on the electrical properties of CSF-TENG and the high electrical output performance of the optimal device is evaluated. Most importantly, the CSF-TENG-based HMI is successfully demonstrated in practicing letters and correcting the writing steps. Finally, 3D virtual reality (VR) applications including letters writing and healthcare based on the CSF-TENG-based HMI are achieved.

## Materials and Methods

### Materials

Sodium Carbonate, lithium bromide, and phosphate buffer (PBS, pH = 7.4) were purchased from Sinopharm Chemical Reagent Co., Ltd., China; EDC hydrochloride was obtained from BOSF Biotechnology Co., Ltd; trypsin (EC 3.4.4.4) and lysozyme (EC 3.2.1.17) were supplied by Shanghai Macklin Biochemical Co., Ltd; deionized water was obtained from a ULUPURE water system; carboxymethyl chitosan was purchased from Aladdin Reagent Company, and raw silks were obtained from the laboratory.

### Preparation of Silk Fibroin (SF)

Raw silk was boiled in a 0.02 M Na_2_CO_3_ solution at 98 °C for 30 min and washed thoroughly with deionized (DI) water to remove impurities and wax; the purified products were submerged in a 9.3 M LiBr solution with a mass ratio of 1:20 at 60 °C. Further, the solution was collected and dialyzed (*M*_*w*_ = 3500) to eliminate residues, and a silk fibroin solution was obtained and denoted as SF.

### Preparation of CSF Film

To achieve a uniform solution, 1 g of carboxymethyl chitosan was dissolved in 25 mL of DI water through stirring. After stirring, the obtained SF was added to the as-formed solution, and 1 mL of EDC hydrochloride (concentration: 30% of the amount of carboxymethyl chitosan) was subsequently added to the solution dropwise after stirring. Further, the mixture was dried in an oven at 60 °C, and the final (CSF) film was obtained. Following the same process, CSF films prepared with a different mass ratio of SF were abbreviated as CSF 0:1, CSF 1:1, and CSF 2:1.

### Fabrication of CSF-TENG

The as-prepared CSF film with a size of 5 × 5 cm^2^ was selected as one electrode, and the PTFE film of the same size was attached to the CSF film. Further, the conductive sponge was chosen as another electrode to cover the PTFE film; a dielectric layer of PTFE film (5 × 5 cm^2^) was attached to the surface between the CSF film and conductive sponge.

### Construction of CSF-TENG Writing System

The CSF-TENG writing system was designed as a woven structure to sensitively respond to mechanical inputs during the handwriting process. The writing system was consist of an 8 × 8 pixel overlapping region on the weft and warp side.

### In Vitro Degradation of CSF Film

CSF film was incubated in a 30 mL solution of trypsin and lysozyme in PBS at 38 °C for 11 days. To obtain a uniform solution, 15 mg each of trypsin and lysozyme was dissolved in 30 mL of PBS, and 100 mg of CSF film was added to the resultant solution. The solution was placed on a shaking table, degraded at 38 °C, and shook at 100 r min^−1^. At fixed intervals, 2 mL of the reaction solution was sampled and measured using UV–vis spectrophotometry. The reaction solution was manually changed daily throughout the degradation process to enhance the degradation efficiency.

### Characterization

The microstructures and morphologies of the samples were obtained using scanning electron microscopy (SEM) and a su1510 microscope. Fourier-transform infrared (FTIR) spectra were obtained on a Nicolet iS10 spectrometer (Thermo Fisher). The chemical states of the samples were scanned by x-ray diffraction (XRD, Bruker AXS) under Cu Kα radiation from 10 to 90 °C. UV–vis spectra of the solution were performed by UV-3600 (Agilent, Cary300). An electrometer was used to measure the TENG’s output voltage and current (Keithley 6514).

## Structure and Output Performance

### Fabrication and Characterization of CSF Film

**Fig. 1:**
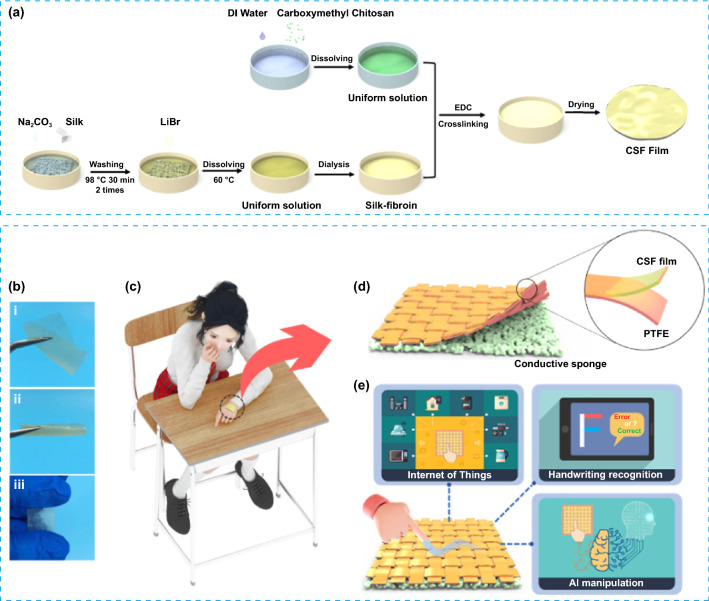
Schematic illustration for preparation process and potential application scenarios. **a** Schematic showing fabrication steps of CSF film. **b** Photograph of twist and stretching of CSF film. **c–e** Schematic diagram of CSF-TENG for HMI, handwriting recognition, and AI manipulation

Figure 1a illustrates the fabrication process of CSF film by crosslinking carboxymethyl chitosan with SF solution. Carboxymethyl chitosan is dissolved in DI water under moderate conditions to form a solution. After adding SF into the resultant solution, EDS hydrochloride is swiftly added while stirring. The formed CSF film is replaced with absolute ethanol and dried at 60 °C after crosslinking. Complex mechanical deformations are measured to intuitively evaluate the physical properties of CSF films. The prepared CSF film can tolerate multidimensional strains under mechanical deformations such as torsional twisting, curl state, and linear stretching, illustrating great potential in wearable sensor, body monitoring and human–machine interaction (Fig. 1b–e).

**Fig. 2:**
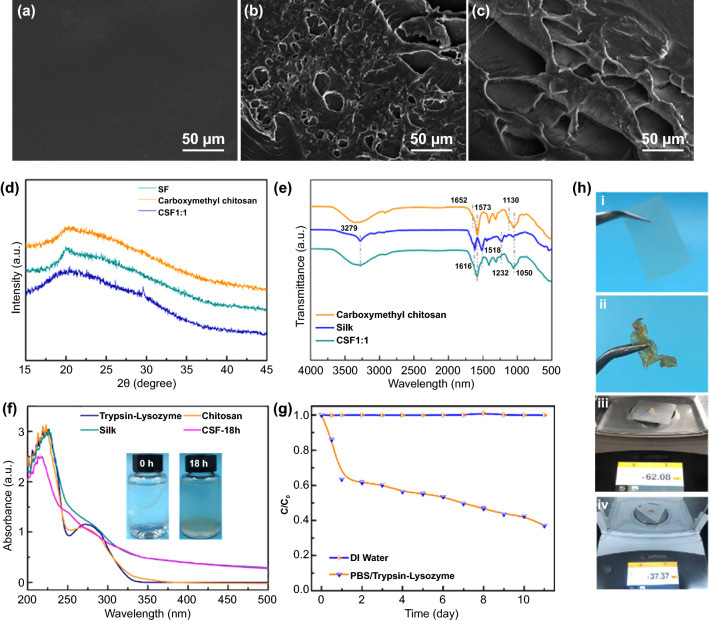
Microscopic images, structure, and biodegradation performance of CSF film. **a–c** Scanning electron microscopic images of SF, carboxymethyl chitosan, and CSF film. **d** XRD spectra of SF, carboxymethyl chitosan, and CSF film. **e** FTIR spectra of SF, carboxymethyl chitosan, and CSF film. **f** Changes of the characteristic absorption after biodegradation by Trypsin-Lysozyme solution for 18 h. Trypsin-Lysozyme and photographs of residual CSF film solution (inset); **g** Biodegradation of CSF film by Trypsin-Lysozyme solution. **h** CSF film and photographs of residual CSF film (**i–ii**); Pictures of CSF film during the biodegradation process (**iii–iv**)

The SEM images in Fig. 2a–c shows the morphological features of SF, carboxymethyl chitosan, and CSF marfilm. Pure SF only has a smooth and dense membrane structure on its surface, and microporous cannot be captured (Fig. 2a). For carboxymethyl chitosan film, the porous structure with open macropores formed by stacking and irregular terraced layered is visible (Fig. 2b). Additionally, the surface of carboxymethyl chitosan displays hills and valleys because of the irregular arrangement of the inner structure. The CSF surface has numerous lacunae within the network and the pore size increases, indicating that the crosslinking SF with carboxymethyl chitosan affects the morphology structure (Fig. 2c).

The crystal phases of all samples were measured using XRD. The XRD pattern of carboxymethyl chitosan shows a broad peak at 20.5° (Fig. 2d), which is consistent with the previous study [65]. A diffraction peak located at 20° is attributed to the amorphous structure of SF [66]. The XRD curve of CSF film retains the diffraction peak of SF and carboxymethyl chitosan after crosslinking but is noticeably different from the two. However, the peak of CSF film weakens and broadens dramatically. This abnormal phenomenon is because of the presence of a high amount of amorphous SF in the CSF film and crosslinking interactions between SF and carboxymethyl chitosan.

FTIR measurements were also performed to adequately account for the interaction structure of SF and carboxymethyl chitosan. Figure 2e shows that the strong peaks located at 1616, 1518, and 1234 cm^−1^ are attributed to the C = O (amide I), N–H (amide II), and C–N&N–H (amide III), respectively. The character peak at 3279 cm^−1^ is assigned to the hydrogen-bonded N–H and O–H stretching vibration. The spectrum of carboxymethyl chitosan shows absorption peaks at 1050 and 1130 cm^−1^, which are typical peaks of the saccharide structure. The amide I and amide II absorption peaks at 1652 and 1573 cm^−1^, respectively, indicate that chitosan has a significant deacetylation degree. Further, the CSF 1:1 film exhibits all the characteristic peaks mentioned earlier, indicating that the SF and carboxymethyl chitosan are effectively crosslinked.

Chemical reagents (NaOH, acid, and H_2_O_2_, etc.) can cause diverse diseases in living things and pose severe threats to ecosystems. Biodegradation is an effective degrading process in which microorganisms or their active by-products (bacteria, fungi, yeast, and polysaccharide) decompose waste in the natural environment [67, 68]. Further, the microorganisms involved in the biodegradation processes generate various enzymes such as protease, hydrolases, and lipases, which directly enhances biodegradation via catalysis. Thus, the biodegradability of CSF was carried out in the mixture of trypsin and lysozyme, which can effectively degrade protein and polysaccharides. The weight loss was measured to determine the periodic decomposition rate of CSF by replacing the enzyme fluid daily. A UV–vis spectrophotometer was used to determine the optical absorption behaviors of silk, carboxymethyl chitosan, trypsin-lysozyme, and CSF degraded for 18 h (CSF-18 h). Figure 2f illustrates that all samples exhibit fundamental absorption in the UV region. The UV spectra of trypsin-lysozyme and carboxymethyl chitosan show a characteristic peak from 250 to 325 nm, corresponding to the formation of π conjugated structure. In comparison with trypsin-lysozyme and carboxymethyl chitosan, CSF’ peak at 250–325 nm disappears after 18 h of degradation, suggesting a catalytic reaction between trypsin-lysozyme solution and CSF. Further, the UV region shows a slight decrease in the CSF absorption intensity, which can be attributed to the hydrolysis of CSF film. However, the inset picture of residual CSF solution confirms that the mixture is increasingly becoming nontransparent because of degradation. Figure 2g shows the degradation rate of DI water and trypsin-lysozyme solution for CSF film. When incubated at 38 °C with constant shaking for 11 days, DI water shows no CSF degradation capacity, whereas, the trypsin-lysozyme solution shows a higher CSF biodegradation ability. The biodegradation rate of CSF film treated by trypsin-lysozyme solution is about 36.53% after 24 h. The degradation rates increase from 36.53% to 63.07% as the reaction time increases, indicating that trypsin-lysozyme plays a positive role in CSF film biodegradation. Figure 2h(i–iv) shows the weight loss of CSF film degraded by the trypsin-lysozyme solution to further validate the biodegradability and environmental friendliness of CSF film; the weight of CSF film noticeably decreases from 100 to 37.37 mg (Fig. 2h(iii–iv)). The result proves that CSF film possesses great biodegradability, and can be ultimately hydrolyzed to tiny molecules.

### Structure and Output Performance of CSF-TENG

**Fig. 3:**
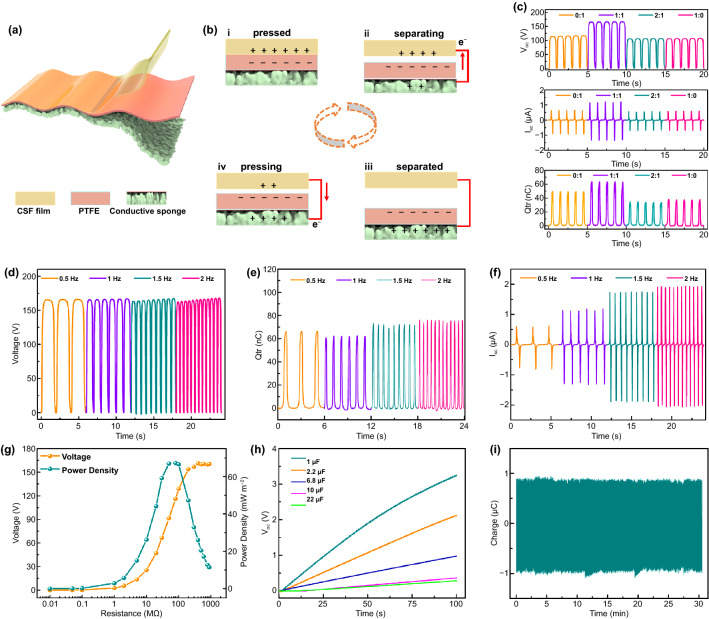
Operating principles and output performance of the CSF-TENG. **a** A schematic structure of the CSF-TENG. **b** A schematic working principle of the contact-separation mode CSF-TENG for energy harvesting. ﻿Electrical output performance of CSF-TENG. **c** *V*_oc_, *Q*_sc_, and *I*_sc_ of CSF-TENG for different introduction concentrations of SF. **d–f** Frequency–response characteristics of CSF-TENG under different loading frequencies (0.5–2 Hz), including **d** *V*_oc_, **e** *Q*_sc_, and **f** *I*_sc_. **g** The variation of voltage and peak power density under varied external resistances. **h** Charging performance under different capacitors (1–22 µF). **i** Stability test of CSF-TENG under continuous impact for 30 min

However, the CSF film is also a high-performance conductive material. A flexible contact-separation mode CSF-TENG is designed after the CSF film is used as an upper electrode (Fig. 3a). Figure 3b shows the electricity-generation principle of CSF-TENG, which uses a typical contact-separation mode. PTFE is selected as a reference material throughout the experiments to determine the electric output performance of CSF film since it is one of the most available negatively charged materials. A piece of conductive sponge attached to PTFE and CSF film is used as back electrodes, which are connected to the external circuit for evaluating the electric output. When the PTFE is touched the CSF film in the original mode, charges are generated from the triboelectrification between the PTFE and CSF film with equally opposite polarities distributed on their surface (Fig. 3b(i)). A potential difference between the conductive sponge and CSF film is formed after the CSF and the PTFE film are separated. Electrons travel from the top of the CSF film to the bottom of the conductive sponge through the external circuit, resulting in an instantaneous current flow (Fig. 3b(ii)). When the PTFE and CSF films are completely separated, an electrostatic equilibrium is achieved between them and the electrons stop transferring (Fig. 3b(iii)). When the CSF film begins making contact again, electrons flow back from the bottom conductive sponge to the CSF film to balance the electrical potential differences (Fig. 3b(iv)). There are no remaining electrons on the electrode when the two charged surfaces fully overlap again, and the CSF-TENG reverts to the original state (Fig. 3b(i)). The electrostatic potential difference of CSF-TENG in the contacting and separating states is further illustrated by numerical calculations (Fig. S1).

A series of CSF films with different amounts of SF (2, 1, and 0.5 g) were fabricated to obtain a high triboelectric patch. The effect of the amount of SF on the electrical output performance of CSF-TNEG was evaluated under mass ratio (CC: SF) ranging from 0:1, 1:1, 1:2, and 1:0 (Fig. 3c). The results show that the CSF 1:1 has the highest electrical output performance (140 V, 1.32 μA, and 64 nC), whereas the output performance of CSF 1:2 and CSF 0:1 gradually deteriorates. Further, the electrical output of the optimized CSF-TENG was accessed by pressing and releasing cycles under various frequencies (0.5–2 Hz), resulting in a maximum *V*_oc_, *Q*_sc_, and *I*_sc_, for 165 V, 77 nC, and 2 μA, respectively (Fig. 3d–f). Further, we will like to investigate how different pressures affect electrical output performance. The *V*_oc_, *I*_s_c, and *Q*_sc_ of CSF-TENG are measured under various pressures (0.65–12.67 N) for verification. Figure S2 shows that the entire electrical outputs are increased linearly as the pressure increases from 0.65 to 12.67 N. This phenomenon is attributed to the increased pressure, which can enlarge the contact-separation area, resulting in higher electrical outputs. The peak voltage was measured as different external resistances to obtain additional details on the behavior of the electrical energy output under external load (Fig. 3g). At a resistance of 1 GΩ, the voltage was at about 165 V, whereas the output power density peaks was at 72 mW cm^−2^. However, the CSF-TENG was successfully used to charge different capacitors at a frequency of 1 Hz. The charging rate increases with decreasing capacitances (Fig. 3h). By manually patting the CSF-TENG, 100 commercial LEDs can be easily illuminated (Fig. S3). For a systematic investigation, an additional test was performed to evaluate the stability of CSF-TENG. Figure 3i shows that the Qsc does not deteriorate after 30 min, demonstrating the strong stability of CSF-TENG. However, pressure is a crucial role in measuring the accuracy of the CSF-TENG in the sensor system.

## CSF-TENG-Based HMI for Letters Practice and Correction

**Fig. 4:**
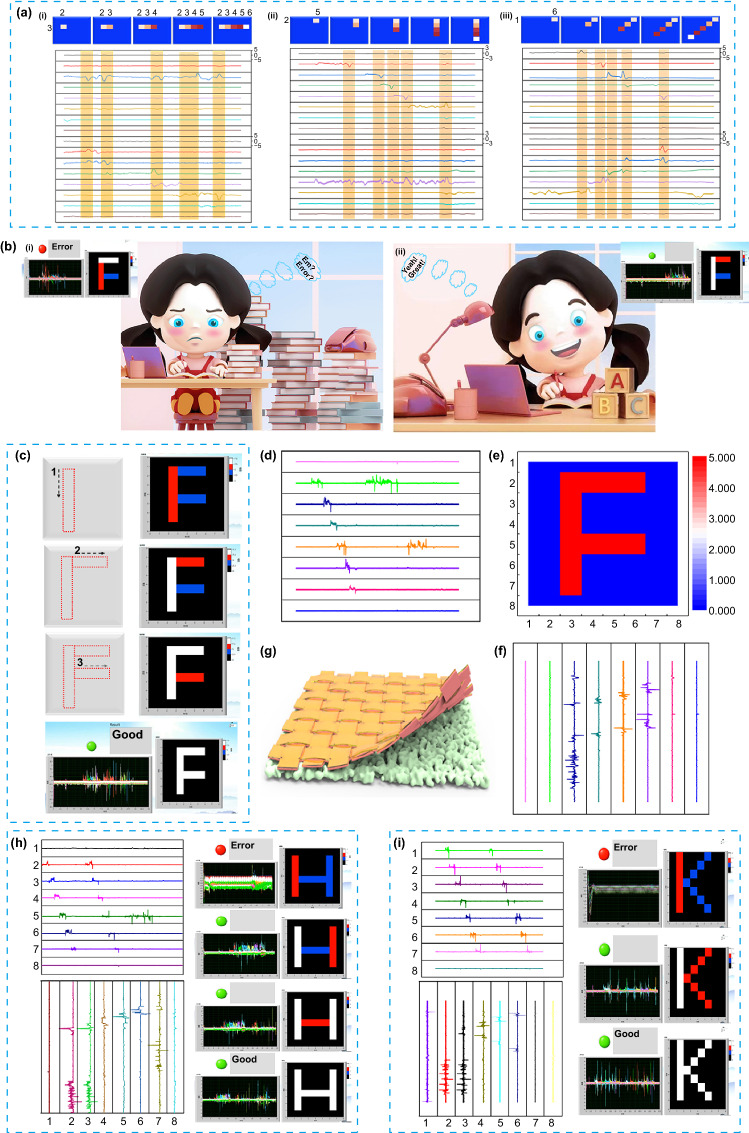
The illustration of details recorded by using CSF-TENG. **a** (**i**) Electrical signals of stroke “ − ” inset: the detailed steps and the output of stroke “ − ” on writing pad. (**ii**) Electrical signals of stroke “|” inset: the detailed steps and the output of stroke “|” on writing pad. (a-iii) Electrical signals of stroke “/” inset: the detailed steps and the output of stroke “/” on writing pad. “1,2,3,4,5,6” represent electrodes. **b** The illustration of the writing results of letter F over different step. **c** The standard of writing steps of letter F and the schematic illustration of letter F recognition and identification process by CSF-TENG-based HMI. **d–f** Signals for writing the letter “F”. **g** Structure and fabrication of the CSF-TENG. **h–i** Signals for writing the letter “H”and “K, the schematic illustration of letter “H”and “K recognition and identification process by CSF-TENG-based HMI

The CSF-TENG-based HMI, which has real-time response capability can be used as an intelligent writing pad to practice and correct letters. It must include a single isolated friction electrode uniformly distributed onto the PTFE to sensitively respond to mechanical inputs during the handwriting process. Thus, CSF mesh fabricated by CSF strips (width: 2 mm) acts as the upper triboelectric layer because of its woven structure. Each point’s output signal is recorded with 2 channels, and varying contact points are connected to 16 channels (Figure S4, supporting information). Figure 4a(i–iii) shows the real-time output signals of the letter writing for different strokes (“**–**,” “**/**,” and “**|**”) obtained by the acquisition card. For the stroke “**–**,” the user briefly touches the first point of the stroke array, and proceeds from electrode 2 to 6, where an output voltage pulse with the regular magnitude is first produced on electrode 2, and then the output voltage of various magnitudes are rapidly generated on electrodes 3, 4, 5, and 6 until the user completes the strokes array without discovering an output voltage (Fig. S5). Because of the real-time response-ability, it is obvious that no matter what kind of strokes the user writes, the handwriting signals and tracks exhibit instant response in the time domain. We selected three letters that are composed of strokes “**–**,” “**/**,” and “**|**” in all letters as an exhibition (“F,” “H,” and “K”). Leveraging the various output information from CSF-TENG, labview is utilized to analyze the signals to identify and correct letters. Figure 4b is the identification and correction images of letter F. The accurate writing standard is given before writing in the case of letter F (Fig. 4c), and it is divided into “**|,**” “**–,**” and “**–.**” The correlation judgment of the letter F is performed based on the standard. A real-time trend can be observed in the voltage on F writing from the output signals (Fig. 4d–h). During the whole process, visible signals are generated as the pressure is applied, and steps corresponding to the motions are performed promptly (Fig. 4d, f and Movie S1), followed by identifying the completed step and making the precise judgment based on the information of letters collected from the CSF-TENG. However, the writing results can be sent back to the user in real-time, allowing them to rewrite the selected letter in the CSF-TENG for validation and amendment. Thus, the corresponding results above the related strokes can be instantly recognized by labview for revision purposes. Although the user’s stroke sequence differs from the given standard, the alert system shows an error warning (Fig. 4b(i)). However, the finished stroke trend is unsatisfactory, deviating significantly from the standard version. For example, when the first writing stroke is “**|**,” the system switches on the green light (Fig. 4b(ii)), whereas an error alert is displayed when the stroke “**–**” is written first. However, when the next red stroke appears, the user should follow the hint to describe it, which will be replaced by a white stroke that is synchronized with the screen’s actual movement. Figure 4c illustrates that the letter F is correctly revised and identified.

Apart from letter F, letters H and K are also explored as representative letters by CSF-TENG-based HMI to make our writing applicable to the normal letters. Figure 4h(i) and Movies S2 and S3 show the images and signals of the last two letters. Figure S5 shows the writing steps of letters H and K that serve as the standards of the two letters. Similarly, when a user writes on the first step of the pad, the stroke gradually generates a distinguishable electrical signal. The images show a distinct sharpness and pattern. As we can see, it is not easy to write letter K smoothly because of the stroke “ < ”. However, the results verify that no matter how difficult the stroke “ < ” it is, CSF-TENG-based HMI can write perfectly and respond immediately. For the two letters, the same conclusion can be drawn: CSF-TENG-based HMI exhibites high rearrangement and correction accuracies. The compelling results also suggest that CSF-TENG-based HMI illustrates perspectives about the future development trends of the portable electronics and workbook practice board, such as letter identification, intelligent revision, and magic calligraphy practice board in the AI/IoT era.

## CSF-TENG-Based HMI for Different Applications

**Fig. 5:**
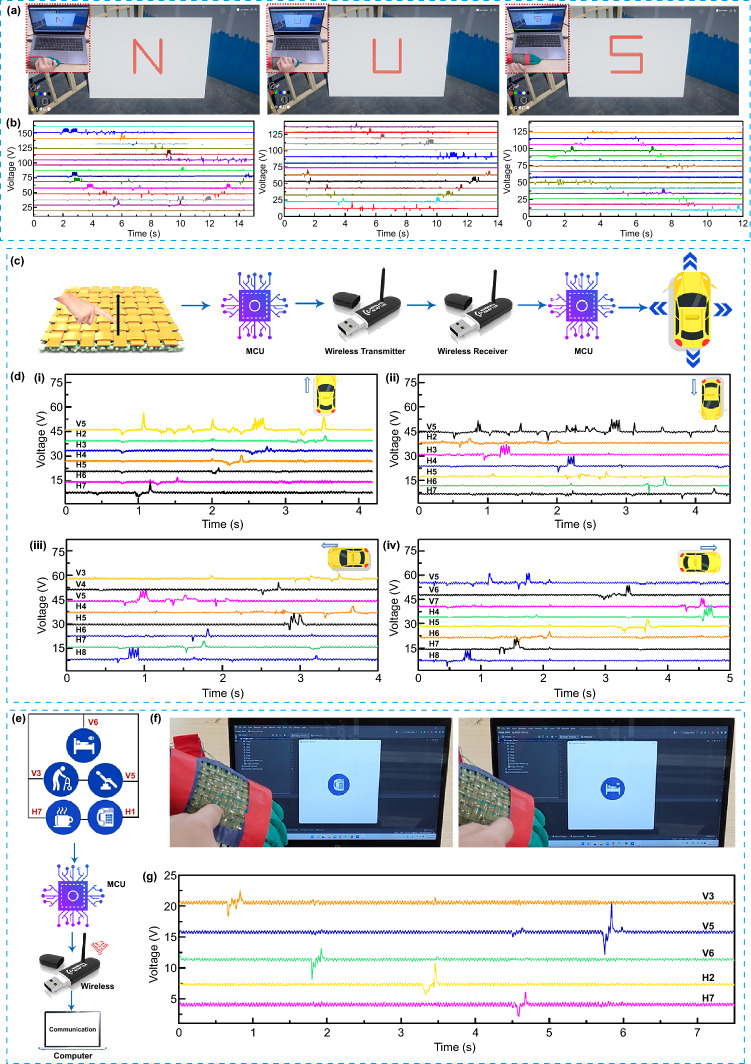
Different applications of CSF-TENG-based HMI. **a** The corresponding screenshot of using steps to achieve VR control in Unity 3D, and the photographs of writing steps of 3 letters (inset). **b** The signal patterns of CSF-TENG corresponding to the three letters. Schematic illustration and real-time signals to control the vehicle by CSF-TENG. **c** Schematic illustration of CSF-TENG-based HMI for vehicle control. **d** The signal patterns of CSF-TENG corresponding to the four directions (**i–iv**). The corresponding movements of vehicle including forward/backward control, and leftward/rightward control (inset). Wearable wireless communication board. **e** Circuit connection of the wireless communication board system. **f** The corresponding icons displayed on the screen and **g** real-time signals when the icons were pressed. (V and H represent vertical and horizontal channel, for example, vertical channel 5 and horizontal channel 5 are abbreviated as V 5 and H 7, respectively)

The flourish advancement of VR and AR technologies provide a creative way for the potential application in social media and personal engagement. Thus, we designed a training program in Unity 3D to verify a VR writing control (Movies S4-S6). Each sensor channel is connected to Arduino for data acquisition. Python processes recieved data in real-time manner and then sends motion command to Unity 3D. Unity 3D receives the command of the predicted steps and then convert it into the movements of virtual pen, as shown in Fig. 5a. In our demonstration, three letters are available for recognition to control the virtual pen, including the letter N, U, and S. When the user writes letters, the steps will be simultaneously synchronized onto the virtual space. Next, the virtual pen in Unity 3D responds to the corresponding order in the virtual board. The triboelectric outputs and VR demonstrations of the three letters are shown in Fig. 5b and Movie S4. Further, the CSF-TENG is also implemented to follow a set of movement routes to prove the HMI for vehicle control, where the arrows represent forward movement, backward movement, leftward-movement and rightward-movement, respectively. The schematic illustration for the vehicle manipulation is shown in Fig. 5c. Simply, the output signals are captured and process by MCU. After, the signals are transffered into digital signals and then detected by the second MCU, which transmit commands to realize vehicle control. In a typical interactive process, the CSF-TENG outputs are employed to control the direction of vehicle movement by tracking the sliding trace of finger. For example, when the finger slides from the bottom to the top, the CSF-TENG from vertical channel 5 to horizontal channel 7 generate electrical signals, thus operating the vehicle to move forwards, backwards, and leftwards as well as rightwards. Figure 5d(i–iv) summarize the output details in responses to the corresponding traces. And the real-time control for vehicle movement commanded by CSF-TENG is intuitively illustrated in Movie S7. Afterward, to provide a simple and intuitive communication command for the special population include the patients and the elderly, the five pixels labeled as drink, lying down, sitting up, emergency contact, and rehabilitation training are presented. Figure 5e illustrates the circuit connection for the communication board. Firstly, the output signals collected from the CSF-TENG convert into digital signals to be transferred out through wireless module. Then the acquired signals are processed and sent to computer based on the MCU. As illustrated in Fig. 5f, a corresponding requirement is displayed on the screen of computer as pressing the emergency contact. Similarly, as the finger touches to the second pixel, the related icon appears verifying real-time and accurate communication (Movie S8). Each simple touching can generate a peak voltage (Fig. 5g). These demonstrations verify the potential of wearable electronics for the realization of the advanced multifunctional HMIs.

## Conclusion

In conclusion, a wearable electronic for letters exercise and automatic correction is developed by CSF-TENG-based HMI. It is implemented by combining TENG and advanced HMI-based real-time data processing. A flexible and wearable CSF-TENG with biodegradable CSF triboelectric film is proposed using the simple assembly method. Contrasting experiments to optimize the output capacity of CSF-TENG are performed, followed by a series of performance measurements. A maximum voltage of 165 V and output power density of 72 mW cm^−2^ at a resistance of 1 GΩ is produced. Further, by using CSF-TENG-based HMI for real-time feature response, the writing process and sequence for the three letters can be tracked and reminded in a timely manner, demonstrating the identification and revision abilities of the proposed system for letters with different shapes. The error warning of the writing result can be generated by writing the same letters with different strokes. It only needs to be lightly touched once in the testing experiment because of its numerous advantages like portability, sensitivity, and rapid response properties. Finally, 3D VR controls including writing, healthcare and vehicle monitoring are successfully displayed using the constructed CSF-TENG-based HMI. Looking forward, an intelligent lifestyle can be established via wearable electronics, automatic identification, correction, and VR applications as a prospect under the HMI and AI infrastructure by employing sensory interactive system.

## Supplementary Information

Below is the link to the electronic supplementary material.

Supplementary file1 (MP4 1766 KB)

Supplementary file2 (MP4 3060 KB)

Supplementary file3 (MP4 2942 KB)

Supplementary file4 (MP4 12094 KB)

Supplementary file5 (MP4 9402 KB)

Supplementary file6 (MP4 14673 KB)

Supplementary file7 (MP4 10654 KB)

Supplementary file8 (MP4 11969 KB)

Supplementary file9 (PDF 1 KB)
