# Supplementary material for: Human Machine Interface with Wearable Electronics Using Biodegradable Triboelectric Films for Calligraphy Practice and Correction
Source: Nanomicro Lett. 2022 Nov 15;14:225. doi: 10.1007/s40820-022-00965-8 (PMC9666580; doi:10.1007/s40820-022-00965-8)
Supplement: Supplementary file 9 — Supplementary file9 (PDF 1 KB) [file 40820_2022_965_MOESM9_ESM.pdf]

Supporting Information for

Human Machine Interface with Wearable Electronics Using Biodegradable Triboelectric Films for Calligraphy Practice and Correction

Shen Shen [1, 2, 3, 4, #], Jia Yi [1, #], Zhongda Sun [2], Zihao Guo [1, 2], Tianyiyi He [2], Liyun Ma [1], Huimin Li [3, 4], Jiajia Fu [3, 4, *], Chengkuo Lee [2, *], Zhong Lin Wang [1, 5, *]

[1] CAS Center for Excellence in Nanoscience, Beijing Key Laboratory of Micro-Nano Energy and Sensor, Beijing Institute of Nanoenergy and Nanosystems, Chinese Academy of Sciences, Beijing 100083, P. R. China

[2] Department of Electrical and Computer Engineering, National University of Singapore, 4 Engineering Drive 3, Singapore 117576, Singapore

[3] Jiangsu Engineering Technology Research Centre for Functional Textiles, Jiangnan University, No.1800 Lihu Avenue, Wuxi, P. R. China

[4] China National Textile and Apparel Council Key Laboratory of Natural Dyes, Soochow University, Suzhou 215123, P. R. China

[5] School of Material Science and Engineering, Georgia Institute of Technology, Atlanta, GA 30332-0245, USA

[#] Shen Shen and Jia Yi contributed equally to this work.

* Corresponding authors. E-mail: kathyfjj@126.com (Jiajia Fu); e0931069@u.nus.edu (Chengkuo Lee); zlwang@mse.gatech.edu (Zhong Lin Wang)

Supplementary Figures

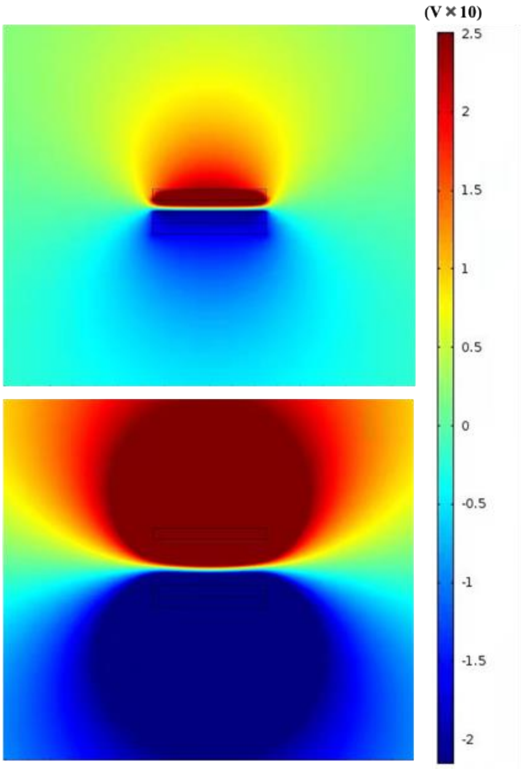

Fig. S1 Numerical calculations of the potential distribution of the CSF-TENG at the different states using COMSOL software

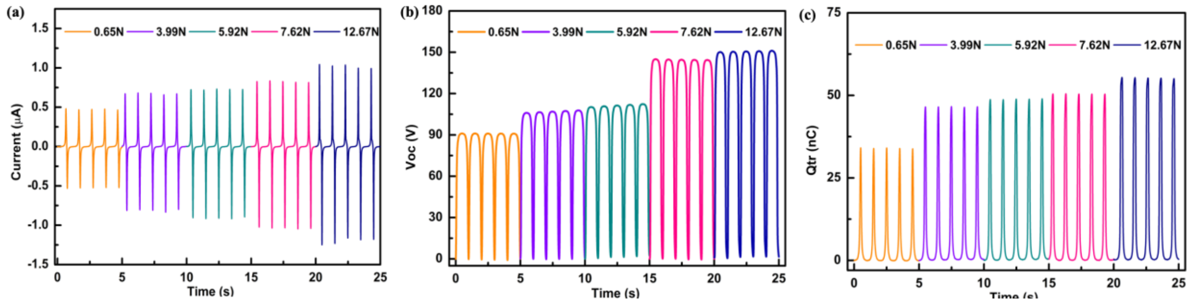

Fig. S2 (**a**) I_{sc}, (**b**) V_{oc}, and (**c**) Q_{sc} of CSF-TENG under varied pressures (0.65−12.67 N) at the frequency of 1 Hz

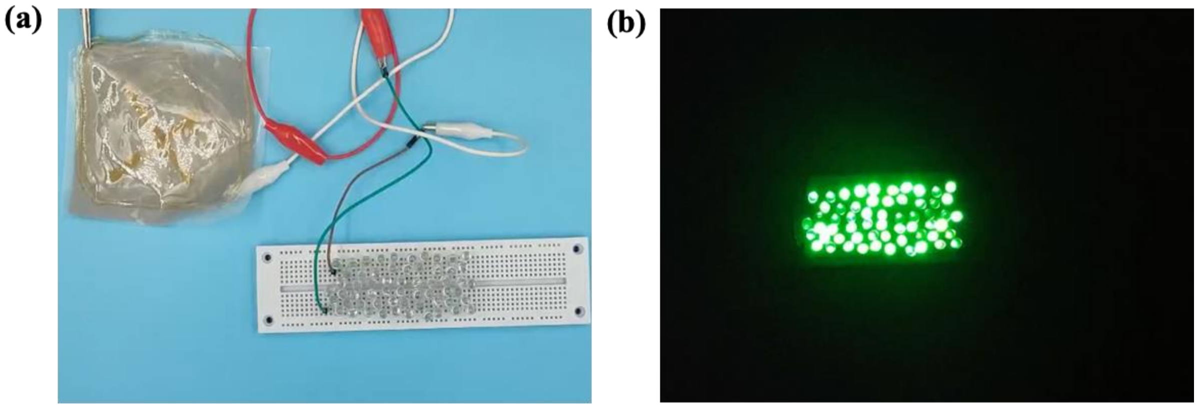

Fig. S3 Images of 100 LEDs lit up using the CSF-TENG, including the overall photo (**a**) and the 100 lighted LEDs (**b**)

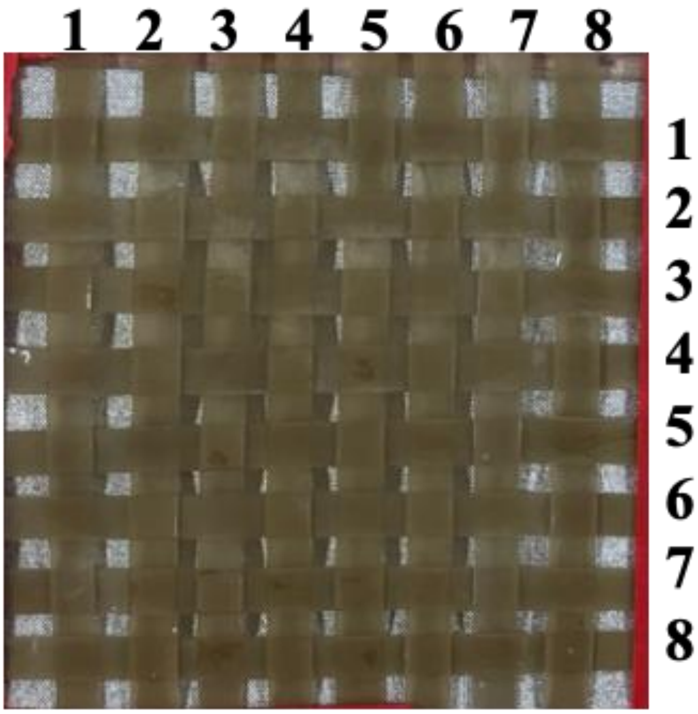

Fig. S4 Photograph of writing pad

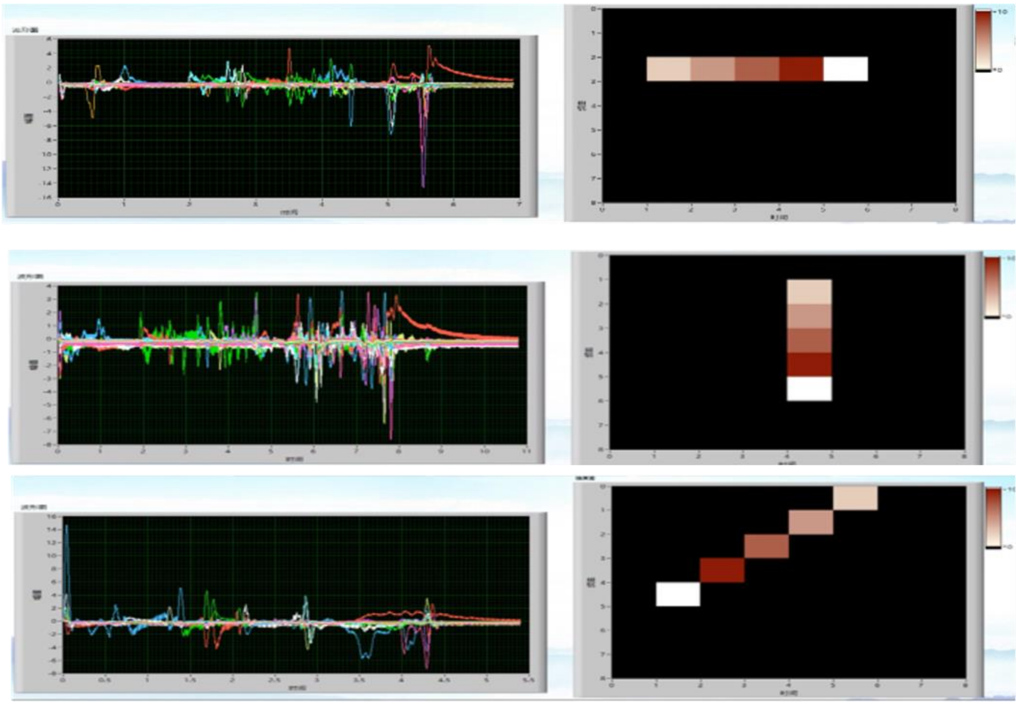

Fig. S5 Schematic illustration of steps "–", "|", and "/" recognition and identification process captured by CSF-TENG-based HMI

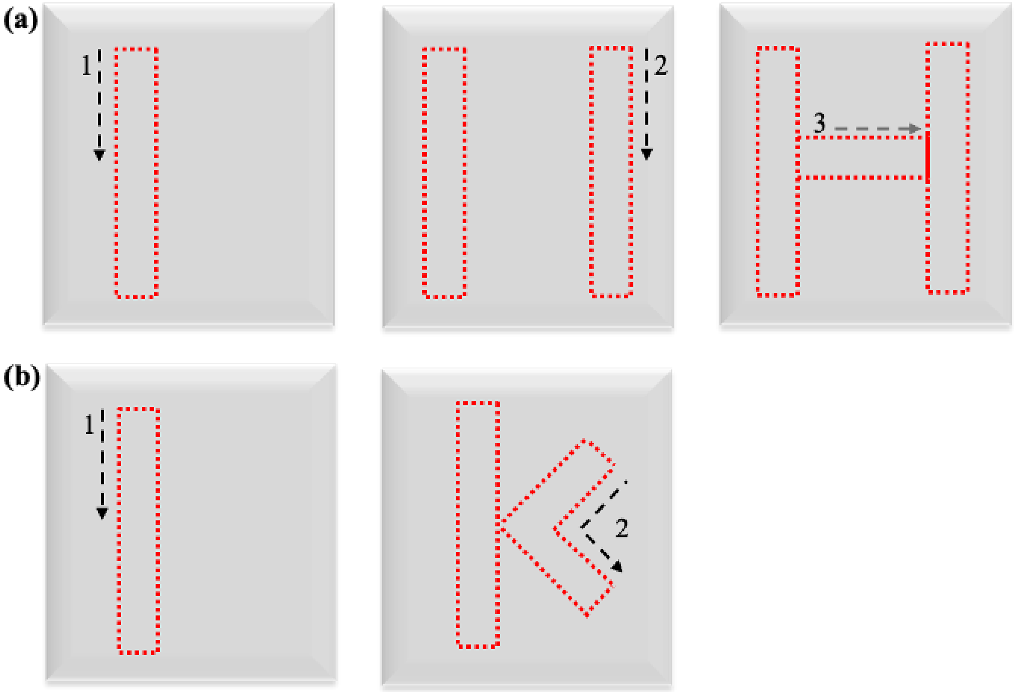

Fig. S6 Standard of writing steps letters. H (**a**) and K (**b**)
